# What do we know about dynamic glucose-enhanced (DGE) MRI and how close is it to the clinics? Horizon 2020 GLINT consortium report

**DOI:** 10.1007/s10334-021-00994-1

**Published:** 2022-01-15

**Authors:** Mina Kim, Afroditi Eleftheriou, Luca Ravotto, Bruno Weber, Michal Rivlin, Gil Navon, Martina Capozza, Annasofia Anemone, Dario Livio Longo, Silvio Aime, Moritz Zaiss, Kai Herz, Anagha Deshmane, Tobias Lindig, Benjamin Bender, Xavier Golay, the GLINT consortium

**Affiliations:** 1 Department of Brain Repair and Rehabilitation, UCL Queen Square Institute of Neurology, Faculty of Brain Sciences, University College London, London, UK; 2 Centre for Medical Image Computing, Department of Computer Science, University College London, London, UK; 3 Institute of Pharmacology and Toxicology, University of Zurich, Zurich, Switzerland; 4 Neuroscience Center Zurich, Zurich, Switzerland; 5 School of Chemistry, Faculty of Exact Sciences, Tel Aviv University, Tel Aviv, Israel; 6 Molecular Imaging Center, Department of Molecular Biotechnology and Health Sciences, University of Torino, Torino, Italy; 7 Institute of Biostructures and Bioimaging (IBB), National Research Council of Italy (CNR), Torino, Italy; 8 Magnetic Resonance Center, Max Planck Institute for Biological Cybernetics, Tübingen, Germany; 9 Neuroradiology, University Clinic Erlangen, Friedrich-Alexander Universität Erlangen-Nürnberg (FAU), Erlangen, Germany; 10 Department of Biomedical Magnetic Resonance, University of Tübingen, Tübingen, Germany; 11 Department of Diagnostic and Interventional Neuroradiology, University Hospital Tübingen, Tübingen, Germany

**Keywords:** MRI, Cancer, glucoCEST, DGE MRI, Glucose, CEST, 3-Oxy-methyl-d-glucose

## Abstract

Cancer is one of the most devastating diseases that the world is currently facing, accounting for 10 million deaths in 2020 (WHO). In the last two decades, advanced medical imaging has played an ever more important role in the early detection of the disease, as it increases the chances of survival and the potential for full recovery. To date, dynamic glucose-enhanced (DGE) MRI using glucose-based chemical exchange saturation transfer (glucoCEST) has demonstrated the sensitivity to detect both d-glucose and glucose analogs, such as 3-oxy-methyl-d-glucose (3OMG) uptake in tumors. As one of the recent international efforts aiming at pushing the boundaries of translation of the DGE MRI technique into clinical practice, a multidisciplinary team of eight partners came together to form the “glucoCEST Imaging of Neoplastic Tumors (GLINT)” consortium, funded by the Horizon 2020 European Commission. This paper summarizes the progress made to date both by these groups and others in increasing our knowledge of the underlying mechanisms related to this technique as well as translating it into clinical practice.

## Introduction

Cancer is a devastating disease which accounts for 16.7% of all deaths worldwide and its early detection is vital to increase the chances of survival (https://www.who.int/health-topics/cancer). Presently, cancer is detected, staged, and followed-up radiologically, through computed tomography (CT), magnetic resonance imaging (MRI), or positron emission tomography (PET). In particular, for over 30 years, diagnosis in oncology has exploited the elevated glucose uptake of tumors by using PET in combination with 2-deoxy-2-^18^F-Fluoro-d-glucose (FDG). It relies on the so-called Warburg effect, characterized by the fact that tumor cells preferentially uptake glucose (or structural analogs) over normal cells, as they rely on enhanced aerobic glycolysis for their energy supply through early metabolic reprogramming [1–4]. Based on the relative uptake of tissues, FDG-PET has enabled the distinction of areas of active tumor from non-tumor or necrotic regions. Furthermore, FDG uptake has been correlated with tumor grade in a wide range of cancers, with an intense PET signal associated to fast proliferating malignant cells [5]. Yet, the technique has its limitations. The radiation exposure restricts repeated scans as well as excludes certain patient groups. Currently, there is an urgent need for an effective and accurate metabolic imaging technique for the detection of new or recurrent tumors and monitoring of treatment response.

### Glucose as a contrast agent for MRI: the hopes, the hype, and the facts

Chemical exchange saturation transfer (CEST) is an MRI method that enables indirect detection of exchangeable amide, amine, or hydroxyl proton groups in small concentrations [6, 7]. In particular, dynamic glucose-enhanced (DGE) MRI is based on glucose uptake through its exchangeable hydroxyl proton groups by CEST (glucoCEST) or glucose-based chemical exchange-sensitive spin-lock (glucoCESL). In the latter case, it will be referred to as T_1ρ_-based DGE (DGE_ρ_) MRI, hereafter. Finally, there are several ways to calculate the signal enhancement, either based on the so-called magnetic transfer ratio asymmetry (MTR_asym_) method, in which case it will be referred to as “mDGE” MRI, hereafter. Note that there is a general trend, when moving from early clinical studies toward more recent clinical ones, to no longer report the difference in MTR_asym_, but to calculate the changes from baseline on a single frequency reference downfield from the water peak. Generally, mDGE MRI seems to be less sensitive than single frequency reference assessment which will be referred to as DGE/DGE_ρ_ MRI for a dynamic acquisition, while glucoCEST or glucoCESL will be referred for a non-dynamic acquisition, hereafter. List of such abbreviation and acronyms used in the paper is summarized in Table 1.

**Table 1:** List of data acquisition-related abbreviation and acronyms used in the paper

Abbreviation	Definition	Acquisition
glucoCEST	glucose-based chemical exchange saturation transfer (d-glucose only)	Non-dynamic
3OMG-based glucoCEST	3-*O*-methyl-d-glucose-based glucoCEST	Non-dynamic
glucoCESL	glucose-based chemical exchange sensitive spin-lock (d-glucose only)	Non-dynamic
DGE MRI	Dynamic glucose-enhanced MRI (d-glucose only)	Dynamic
3OMG-based DGE MRI	3-*O*-methyl-d-glucose-based DGE MRI	Dynamic
DGE_ρ_ MRI	T_1ρ_-based DGE MRI (glucoCESL; d-glucose only)	Dynamic
MTR_asym_	Magnetization transfer ration asymmetry	Dynamic or Non-dynamic
mDGE MRI	MTR_asym_ based dynamic glucose-enhanced MRI (d-glucose only)	Dynamic

DGE MRI has come up as one of the possibly most interesting CEST applications in cancer [8–10]. Preliminary animal data demonstrated a pattern of DGE signal increase using either D-glucose (Glc) [8, 9] (hereafter referred to as “DGE”) or glucose analogs, e.g., 3-O-methyl-D-glucose (3OMG) (hereafter referred to as analog-based DGE, e.g., 3OMG-based DGE) in various tumor models [11, 12] (Table 1). In addition, recent work by several groups showed the possibility of detecting other metabolites based on the CEST effect from hydroxyl protons, such as sucrose, glucosamine (2-amino-2-deoxy-d-glucose, GlcN), and *N*-acetyl-glucosamine (GlcNAc) [13–15]. Thus, developing new MRI techniques with increased sensitivity to substrate levels of non-radioactive glucose analogs holds promise as a potential replacement for the ubiquitous FDG-PET [16], with the caveat of course that FDG is used in tracer quantities, while any MRI contrast agent requires orders of magnitude larger concentrations for it to be detectable (usually in the mM range). Following early animal studies, several groups have started working on the clinical demonstration of the technique. While some demonstrations of enhanced CEST signal in the human brain following the injection of Glc have been shown at 7 Tesla (T) [17–21], the results at more clinical field strengths of 3 T have been less impressive so far [22, 23]. Finally, three studies have attempted applying this technique outside of the brain at 3 T [24–26]. Therefore, development of DGE MRI methodology for clinical use is far from being complete, and many opportunities still remain.

### GLINT consortium

One of the recent international efforts aiming at pushing the boundaries of this translation of the DGE MRI technique into clinical practice has been the glucoCEST Imaging of Neoplastic Tumors (GLINT) consortium, made up of a multidisciplinary team of eight partners and funded by the Horizon 2020 European Commission (2016–2019; http://www.glint-project.eu/). The main aims of that project were to try and assess both Glc and a non-metabolizable glucose derivative (3OMG), used either independently or as a combined examination in clinical radiological oncology practice to assess cancer glucose uptake and metabolism. To establish this new diagnostic in vivo imaging tool, the following main scientific objectives were defined:

I. To improve MRI technologies and image processing tools to be able to better detect the expected small changes in DGE signal at clinical field strength;
II. To establish the compartmental origin of the signal using both Glc and methylated glucose in DGE MRI;
III. To establish detection thresholds and response to therapy in animal models of solid cancers of both native and methylated glucose;
IV. To demonstrate efficacy and safety of the DGE MRI method using Glc through extensive testing in human neoplasms;
V. To assess 3OMG toxicology, bio-distribution and pharmacokinetics in rodents;
VI. To develop an integrated software for the efficient detection and analysis of DGE MRI in the clinic.

As such, this consortium was ideally positioned to push the envelope of the knowledge and technology in this nascent field, and we aim in this paper to review the progress achieved both within and outside this consortium to date.

## Origin of the DGE signal

Despite considerable research effort in identifying the specifics of the DGE contrast, its exact cellular origin is still unclear. While the glucose hydroxyl protons are thought to provide a main source for the DGE signal, other effects might also contribute to a change in the observed signal upon glucose injection. In particular, a sudden increase in arterial osmotic pressure could also trigger large changes in the signal. Such osmotic effect would artificially increase the CEST effect after injection, which can be important if the DGE contrast is acquired without corresponding upfield reference [27]. However, the contribution due to osmolarity changes has been shown to be rather small [28] when using the MTR_asym_ method [29] to calculate the DGE effect (mDGE). Other effects, such as alteration in flow and oxygenation levels in the blood, or even volume changes of blood and CSF, might also contribute to signal changes. However, Walker-Samuel et al. [9] could not detect any changes in pH or cerebral blood flow (CBF), thus excluding direct hyperglycemia-related effects as a source of contrast for DGE MRI. If the blood oxygenation levels were to change, it would lead to a small signal change in the vessels due to the change of T_2_*, similar to the blood oxygenation level dependent (BOLD) effect. Yet, Nasrallah et al. showed a lack of detectable CEST (mDGE) signal after the bolus injection of l-glucose, a glucose analog with identical hydroxyl protons yet limited blood–brain barrier (BBB) permeability [28]. This observation seems to indicate the lack of a vascular contribution to the signal. However, further studies would be needed to fully validate this somewhat counterintuitive finding, given the high levels of l-glucose in the blood compartment after bolus injection.

### In vivo assessment of glucose metabolism at single cell level

In addition to l-glucose, Nasrallah et al. also detected a DGE signal using both Glc and 2-deoxy-d-Glucose (2DG) [28]. As such, the DGE signal was assessed, as part of the GLINT consortium, in this organ model. In particular, the individual contributions of the two main brain cell populations, neurons, and glia to the cerebral DGE signal, as opposed to the extracellular compartment, remained unknown at the start of the project. Knowledge about the underlying cellular origin should therefore help better interpret the DGE data. As part of the GLINT consortium, the combination of fiber photometry and DGE MRI was chosen for a direct comparison of the MR-based glucose measurements with the intracellular variations of the concentration of glucose and its metabolites (e.g., pyruvate). Thus, Eleftheriou et al. [30] performed simultaneous CEST MRI and optical measurements, to assess the intracellular glucose response in the somatosensory cortex of the healthy mouse brain in vivo upon injection of Glc.

**Fig. 1:**
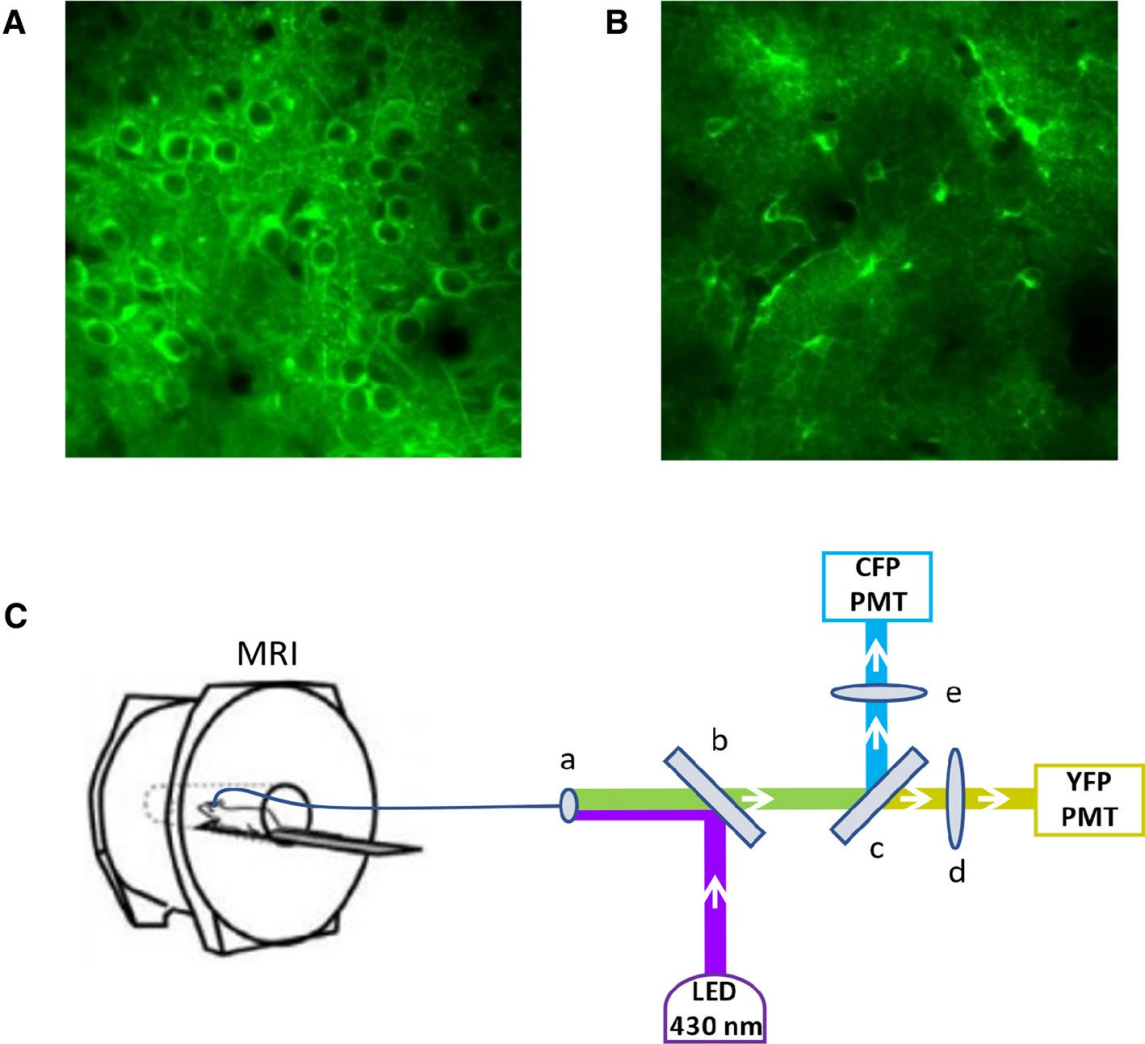
**A** 2PLSM image of neuronal expression of FLIIP, **B** 2PLSM image of astrocytic expression of FLIIP, **C** Experimental setup of simultaneous DGE MRI (7 T Bruker BioSpec 70/30) and fiber photometry measurements: (a) optic fiber connector, (b) dichroic mirror 455 nm, (c) dichroic mirror 515 nm, (d) bandpass filter 530/43 nm, (e) bandpass filter 475/42 nm

As part of these experiments, DGE measurements were performed on a 7 T Bruker BioSpec 70/30 MRI scanner. Recordings of relative intracellular Glc concentration changes were performed using a custom-made fiber photometry setup optimized for Förster resonance energy transfer (FRET) sensors, that require two detection channels and accurate subtraction of the optic fiber autofluorescence. The Glc-dependent fluorescent signal was generated using the genetically encoded nanosensor FLII12Pglu600μΔ6 (FLIIP) [31], which was codon diversified, to overcome homologous recombination problems [32], and expressed in the somatosensory neocortex of adult mice using an Adeno-Associated Viral (AAV) vector. The cell-specific promoters human Synapsin (hSyn) and short Glial Fibrillary Acidic Protein (GFAP) were used to ensure selectivity of expression in neurons and astrocytes, respectively (Fig. 1A, B).

**Fig. 2:**
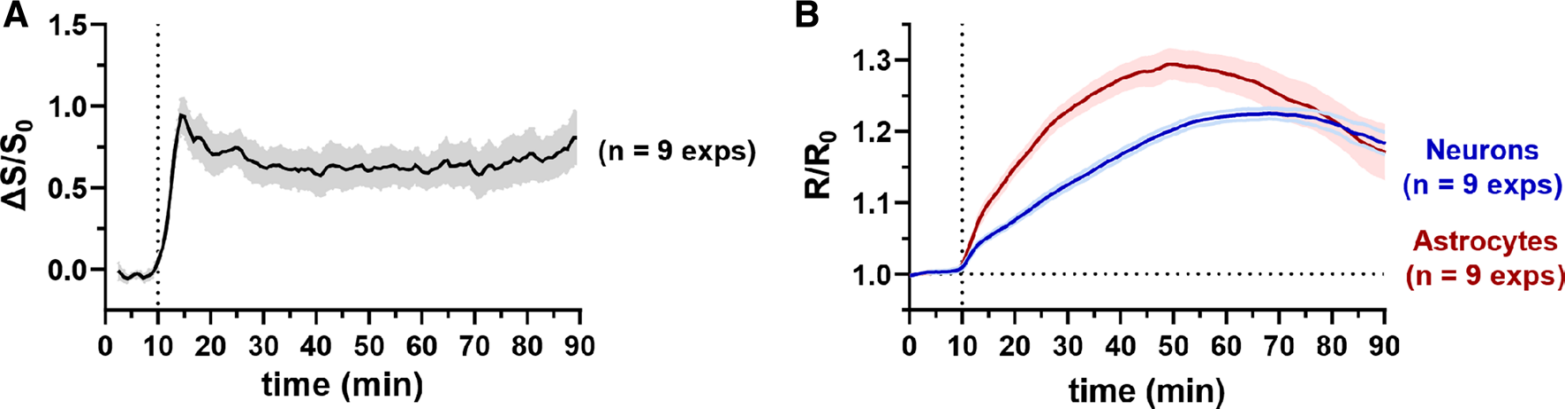
Glucose response curves upon intravenous injection of 120 μl 50% w/v glucose solution at normoglycemia after 10 min baseline. **A** DGE signal, whole brain, 1 mm slice, **B** 2PLSM (λ_exc_ = 870 nm) in astrocytes and neurons, respectively

The feasibility of measuring changes in the FLIIP signal using fiber photometry was validated by comparison with two-photon laser scanning microscopy (2PLSM), using the same Glc injection protocol. Upon injection of 120 μl of a 50% w/v Glc solution intravenously at normoglycemic conditions, Eleftheriou et al. observed a rapid increase in the DGE signal, reaching a maximum within a few minutes and stabilizing at that level for more than an hour (Fig. 2A), in accordance with previous studies [16, 33, 34]. In contrast, 2PLSM showed slower kinetics both in astrocytes and neurons, taking about 40 min to reach a maximum and then progressively decreasing (Fig. 2B). Differences were observed between the two cell populations, with astrocytes reaching the maximum about 10 min faster than neurons. These results show that simultaneous monitoring by fiber photometry is a promising approach to produce both qualitative and quantitative insights on the origin of DGE signals.

In particular, the observed intracellular responses of the two most abundant brain cell types allow to draw important conclusions about the origin of DGE signal. The initial rapid increase in DGE signal is unlikely to be of cellular origin, but seems to predominantly reflect the intravascular and/or extracellular concentration of Glc [8], that is known to respond rapidly to blood glucose concentration changes upon injection of Glc [35]. However, the precise relative contribution of the different compartments (intravascular, extracellular, intracellular) cannot be assessed using this method. Based on a direct comparison between mDGE signal response following Glc and l-glucose administration, Nasrallah et al. [28] hypothesized that the early signal increase in the brain could be of extracellular origin. The remarkable stability of the DGE signal during the raising phase of the 2PLSM traces might be explained by the mix of different extra- and intracellular compartments. In fact, whether blood glucose is detectable or not by glucoCEST, the known progressive decrease in blood glucose levels after bolus injection would be directly translated into a reduction in extravascular extracellular glucose levels. With the ongoing glycolytic activity, the glucose concentrations are expected to decrease everywhere. The lack of a corresponding decrease in DGE signal in these experiments remains therefore difficult to explain.

Based on the present study, it can be concluded that the cerebral DGE signal most likely originates from both the extracellular space and potentially from a mix of intracellular neuronal and astrocytic concentrations, with a relative contribution that dynamically changes over time. These temporal dynamics remain, however, undetectable by DGE MRI itself, due to the lack of specificity of the CEST signal. Moreover, in the experimental conditions applied here, the decrease of intracellular glucose concentration due to its consumption is not reflected in the DGE response. Therefore, while DGE MRI is an excellent technique to observe the localization of injected glucose in different brain areas and tumor tissue, the interpretation of the underlying glucose kinetics remains challenging, and for a large part, unresolved.

### In vivo assessment of glucoCEST signal dependence on extracellular pH

As shown previously, certain aspects of the glucoCEST signal might be related to pH changes. Indeed, the CEST effect is primarily affected by pH [36]. The fast exchange rate of hydroxyl protons might be reduced in an acidic pH environment, leading to an improved detection of the glucoCEST signal at clinical field strengths. As such, the potential influence of the tumor extracellular pH has to be considered in glucoCEST MRI studies. In fact, the majority of cancerous cells exhibit increased glucose uptake and dysregulated glycolysis, with a consequent decrease of extracellular pH, an established hallmark of tumor microenvironment. As part of GLINT, Longo and co-workers have developed several MRI-based approaches for imaging in vivo tumor acidosis [37–40], showing a robust correlation between tumor glycolysis and extracellular acidification [41].

To optimize detection of glucoCEST images in the small animal, a fast and whole volume coverage CEST sequence was developed, using a multi-slice centric reordered single-shot RARE (Rapid Acquisition and relaxation enhancement) acquisition based on uneven saturation irradiation [42]. This sequence exploits a longer saturation period for the first slice and repeated shorter saturation pulses (to maintain a constant saturation) for all the subsequent slices. Consequently, it can provide a superb high spatial resolution (0.23 × 0.23 × 1.5 mm^3^) in animals within the whole tumor by sampling ca. 50 frequencies in less than 10 min.

**Fig. 3:**
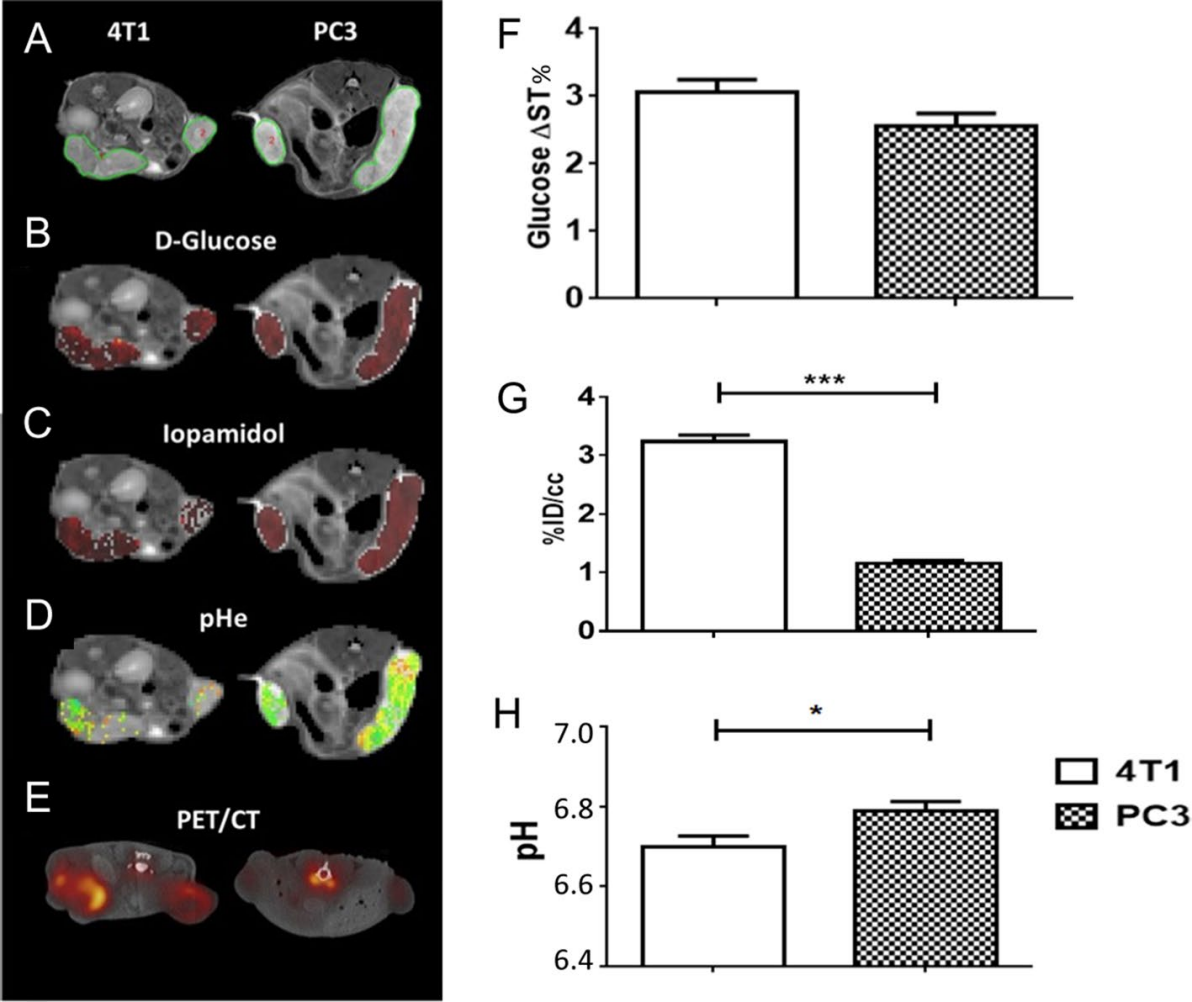
Representative T_2_w images (**A**), DGE map after d-glucose i.v. injection (**B**), CEST contrast (**C**) and tumor pH maps (**D**) after iopamidol I.v. injection, fused ^18^F-FDG-PET/CT images (**E**) for 4T1 and PC3 tumor-bearing mice. Average values calculated for each tumor model of Glucose ΔST% (**F**), ^18^F-FDG PET uptake as %ID/cc (**G**) and tumor pH (**H**)

To provide insights about the in vivo glucoCEST signal dependence with the tumor extracellular pH, Aime’s group combined glucoCEST MRI with tumor pH imaging in two tumor models exhibiting distinct metabolic rates (4T1: murine triple negative breast cancer and PC3: human prostate cancer) by sequential imaging following Glc and Iopamidol injection [43]. Moreover, ^18^F-FDG-PET imaging was performed in the same animals to assess glucose uptake. They observed that 4T1 tumor model displayed a greater glucoCEST contrast and higher ^18^F-FDG uptake in comparison to the PC3 tumors. Consistently with the higher glucose uptake, 4T1 tumor model presented a more acidic extracellular pH than the PC3 model, reflecting an increased tumor acidosis (Fig. 3). In this study, a moderate correlation was observed between tumor acidosis and glucoCEST contrast, with more acidic tumor models showing higher glucoCEST contrast, in line with the expectation of reduced exchange rate for extracellular glucose hydroxyls at low pH [8, 9].

### CEST MRI contrast and environmental considerations

**Fig. 4:**
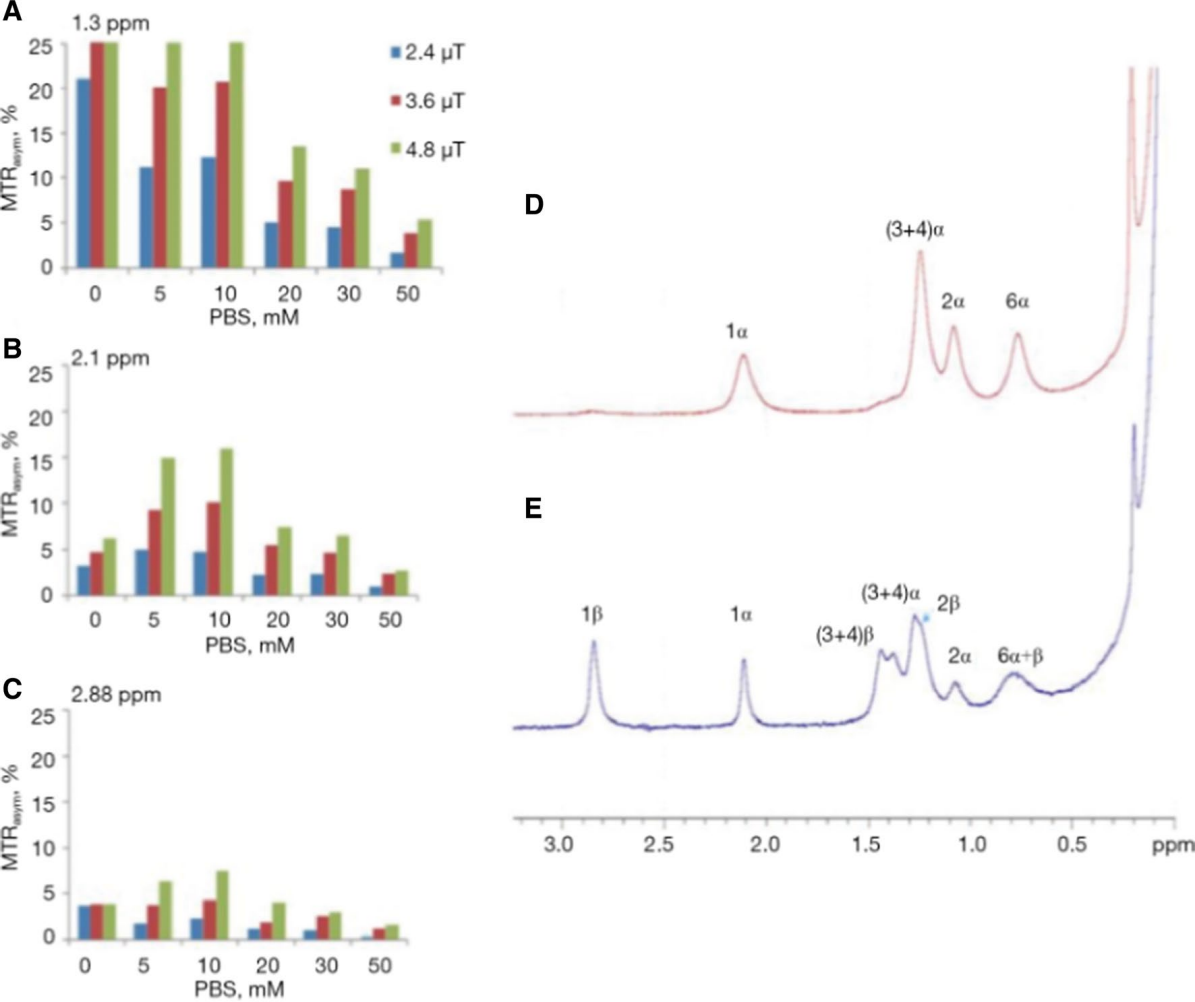
Bar graph showing % of MTR_asym_ of 20 mM glucose solution (10% D_2_O) at several PBS concentrations at the typical frequency offsets of the hydroxyl peaks (**A**) 1.3 ppm, (**B**) 2.1 ppm and (**C**) 2.88 ppm from the water peak (*T* = 37 °C, 11.7 T). ^1^H NMR spectra of the hydroxyl protons of 0.1 M D-Glc solution (*T* = 4 °C, pH = 5.4, 11.7 T). Spectra were recorded on a fresh sample (**D**) and several hours after the sample preparation (**E**). From [44], with permission

The CEST contrast depends on the proton exchange rate of the labile protons with water. However, since proton exchange rates in biological systems depend on many physiological parameters, translation to a clinical setting is challenging. CEST contrast is often affected by the acidity of the detected environment, the type and concentration of buffer solution, etc. An example of the acute effects caused by the change in buffer concentrations is shown in Fig. 4. The drastic changes in the MTR_asym_ plot around the resonance frequency of the hydroxyl peaks due to the buffer change may indicate the difficulty in characterizing the source of the effect. Therefore, in most cases, it should be considered that the CEST contrast represents a contribution from multiple sources [44].

## Glucose and analogs in preclinical animal models

### Comparison between Glc and 3OMG

Sehgal et al. [45] showed the feasibility of using 3OMG as a CEST agent for detecting a malignant human brain tumor (U-87 MG) in a mouse model. They demonstrated that 3OMG shows a CEST contrast enhancement that is approximately twice as much as that of Glc for a similar tumor line. These results are consistent with that obtained for the breast tumor model (4T1) [46] and show the feasibility of using 3OMG as a CEST agent for detecting malignancies.

Both Glc and 3OMG have been investigated in several studies for their CEST contrast. To the best of our knowledge, this is the first study to examine the side-by-side comparison of the two methods in the same animals. For that purpose, mice bearing the 4T1 breast tumor model were scanned in a 7 T scanner using the same CEST protocol on the same day, with an interval of ∼8 h between the two methods to ensure that the CEST signal returned to its baseline level. The results showed that these two agents have different CEST profiles: 3OMG-based DGE MRI showed a higher CEST effect than Glc for the breast cancer model, even at only half the dose used for the latter (0.7 g/kg 3OMG versus 1.5 g/kg Glc). Moreover, the 3OMG-based DGE effect lasted for more than an hour, whereas the DGE effect lasted for only a short time, causing the contrast to disappear completely [46]. That is, the CEST signal of Glc was less stable than that of 3OMG, which is a clear disadvantage in clinical practice.

In addition, Anemone et al. investigated, for the first time, under the same experimental conditions, CEST image analysis and tumor model the CEST contrast efficiency of Glc and 3OMG with the aim of a robust comparison of their properties and CEST efficiency at the two field strengths of 3 T and 7 T [47]. Interestingly, a strong and different pH dependence of the CEST contrast was observed between Glc and 3OMG, with Glc showing higher CEST contrast moving toward acidic pH values (max ST% at pH 6.0–6.2), whereas 3OMG displayed higher CEST effects when the pH was closer to neutral values. Despite the marked differences in pH behavior and metabolic fate of the two molecules, in vivo results in a melanoma tumor murine model showed comparable glucoCEST contrast following intravenous administration. Both Glc and 3OMG provided similar CEST contrast (in the range of 2–3%) at 7 T and at 3 T (range 1–2%) upon i.v. administration with a dose of 3 g/kg. Lower doses resulted in reduced CEST contrast that was lower than 1% at 3 T. Of note, the CEST increase in tumor was almost constant for 3OMG over the 30 min period of observation, whereas a marked increase along time was detected for Glc. This different time-dependent behavior of the CEST contrast suggests unequal contributions from the intracellular and extracellular compartments, linked to the specific metabolic fate and to the observed dissimilar pH dependence of Glc and 3OMG. It also looks like these different concentrations between intra- and extracellular compartments is much different in the brain than in tumors, as shown by Eleftheriou et al. [30]. Moreover, one may surmise that the agent use, in the case of Glc, might directly lead to a reduction in pH due to the enhanced glycolysis as a consequence of the higher availability of glucose to cancer cells. However, in vivo detection at clinical magnetic field strength (3 T) turned out to remain quite challenging for both the molecules and requires accurate parameter optimization [48].

### Comparison of MRI glucoCEST signals to PET for monitoring response to therapy

As mentioned in the introduction, ^18^F-FDG-PET imaging is commonly performed for tumor diagnosis and plays an important role in therapy monitoring [49, 50]. Although glucose and ^18^F-FDG possess a different metabolic fate, with both readily taken up by cancer cells, a good spatial accordance between the ^18^F-FDG autoradiography and glucoCEST images was observed in colorectal tumor murine models in the original glucoCEST investigations [9]. On the other hand, few studies had investigated in vivo relationship between the glucoCEST signal and ^18^F-FDG-PET [45, 46].

**Fig. 5:**
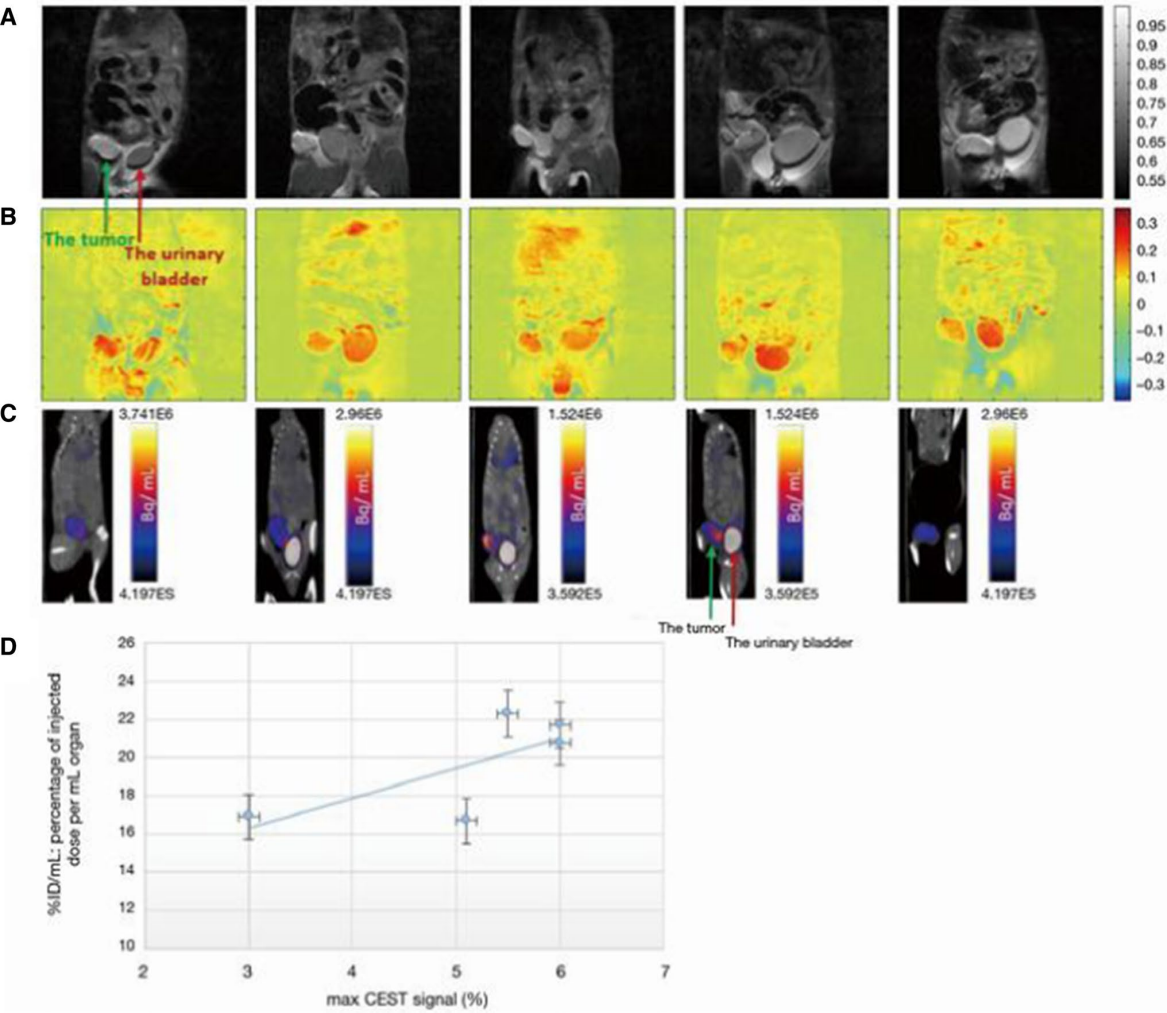
3OMG-based DGE MRI and ^18^F-FDG-PET/CT images from five tumors of a murine model (4T1 cells). **A** A coronal view of an anatomical T_2_-weighted MR images (7 T field) before 3OMG administration showing the tumor (green arrow) and the urinary bladder (red arrow). **B** % CEST images 60 min after *per os* administration with 3OMG, 1.0 g/kg (at a frequency offset of 1.2 ppm, B_1_ = 2.4 µT). A significant CEST contrast was obtained in the tumor and the urinary bladder as well as areas suspected to be metastases. **C** ^18^F-FDG-PET/CT coronal view obtained 60 min after IV injection of ^18^FDG showing accumulation mainly in the tumor (green arrow) and urinary bladder (red arrow). **D** Correlation between 3OMG % CEST contrast and % ID/mL value in the five tumors from a murine model ± S.D [44]. From [44], with permission

In particular, Rivlin and Navon [46] undertook to compare CEST contrast and ^18^F-FDG-PET uptake, following the administration of the 3OMG in a breast murine tumor model (4T1). PET measurements were performed 2 days after CEST-MRI acquisitions, 3OMG was administrated *per os* (1 g/kg), and CEST images were acquired for 60 min after the administration. A similar trend and imaging outcomes were found between two modalities as demonstrated in Fig. 5. From these experiments, a correlation between FDG uptake and 3OMG-based glucoCEST MRI can be established, providing further validation of the use of 3OMG-based glucoCEST MRI as an indicator of glucose uptake.

Regarding therapy monitoring, to date, glucoCEST imaging has been assessed in a glioblastoma murine model for evaluating the treatment response to rapamycin, an inhibitor of the mTOR pathway, but without any comparison with the ^18^F-FDG-PET technique [51]. Consequently, a proper assessment of the glucoCEST technique in evaluating treatment response should be investigated for a potential replacement of the ^18^F-FDG-PET imaging.

Thus, Capozza et al. investigated whether the glucoCEST approach can monitor the metabolic response to anticancer therapies in a breast murine cancer model and compared the results with those obtained with the ^18^F-FDG-PET approach [52]. In this study the metastasizing triple negative breast tumor 4T1-bearing mice were treated for two weeks with i) a conventional chemotherapeutic drug (doxorubicin) or ii) with dichloroacetate (DCA) that targets tumor metabolism by reversing the Warburg effect [53].

4T1 tumor volume was dramatically reduced after three cycles of doxorubicin treatment in comparison to the untreated group, whereas tumor size was not reduced after DCA therapy. In accordance with the tumor volumetric changes, the glucoCEST contrast decreased upon doxorubicin treatment, whereas no changes were observed following DCA administration, in agreement with the lack of effect on tumor size. Of interest, ^18^F-FDG-PET imaging did not report any change in glucose uptake or metabolism for both doxorubicin and dichloroacetate treatments.

Overall, this study indicated a higher sensitivity for glucoCEST imaging in comparison to ^18^F-FDG-PET for assessing the early response to doxorubicin treatment, although additional studies in other cancer types and therapeutic regimens are needed to confirm these findings.

### Specificity of glucose analogs

#### 3-*O*-methyl-glucose

**Fig. 6:**
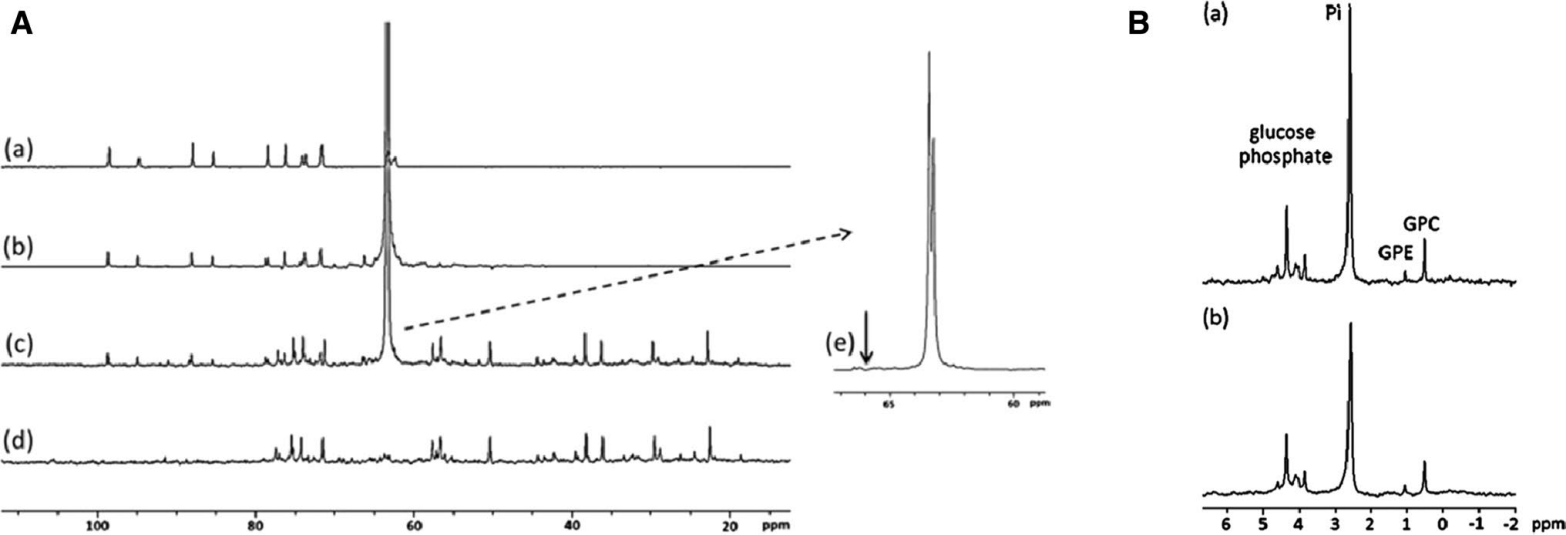
**A** ^1^H‐decoupled ^13^C NMR spectra of 100 mM 3OMG solution (a), 100 mM [6‐^13^C]‐3OMG solution (b), combined extracts from 4T1 tumors treated with [6‐^13^C]‐3OMG (1 g/kg) (c), combined untreated extracts from 4T1 tumors (d), enlargement of (c and e). The arrow represents the expected position of the phosphorylated product of 3OMG (3OMG‐6‐phosphate) on the basis of the observed glucose‐6‐phosphate at 65.6 ppm and the 2‐DG‐6‐phosphate peak at 66.0 ppm. **B** ^1^H‐decoupled ^31^P NMR spectra of combined extracts from 4T1 tumors treated with [6‐^13^C]‐3OMG (1 g/kg) (a), combined untreated extracts from 4T1 tumors (b). GPC‐glycerophosphocholine, GPE‐glycerophosphoethanolamine, Pi‐inorganic phosphate; spectra were calibrated according to GPC (0.49 ppm). From [46], with permission

The potential biochemical pathways and sources of CEST contrast for glucose analogs were evaluated by exploring their uptake and metabolism. Enhanced uptake of glucose or glucose analogs in tumors occurs through overexpression of glucose transporter proteins (GLUTs), which are highly expressed in tumor tissues. Therefore, ^13^C and ^31^P NMR spectroscopy measurements were first performed to analyze the origin of 3OMG signal. The ^13^C NMR spectra of combined extracts from 4T1 tumors after *per os* administration of [6-^13^C] 3OMG (1.0 g/kg) are shown in Fig. 6A. The results indicate the penetration of 3OMG into the tumors (peak at 63.3 ppm), while no other metabolite was observed. The ^31^P NMR spectra of extracts from 4T1 tumors also showed no evidence of phosphorylated products in the treated tumors (Fig. 6B). Both results confirm the generally accepted notion that 3OMG can be considered as a “non-metabolized” glucose analog that enters cells via the membrane concentrating sodium-dependent glucose transporter and exits cells via the membrane-facilitated diffusion transporter [54].

Finally, the absence of 6-phospho-*O*-methyl-d-glucose in the brains of mice after administration of [6-^13^C] 3OMG (1.0 g/kg, PO) was demonstrated by both ^13^C and ^31^P NMR spectroscopy studies of the metabolites extracted from the brains, reinforcing the fact that 3OMG can indeed enter the brains, albeit without any other metabolite as described in Rivlin et al. [44].

#### Glucose analogs with accumulation effects

The two glucose analogs 2DG and FDG are both taken up by cancer cells, undergo phosphorylation, however, without being further metabolized, and thus accumulate in the cells. As such, 2DG and FDG were shown to give enhanced and stable CEST MRI signal when injected in orthotopic rodent models of mammary tumors [12, 28]. These two glucose analogs present, however, with a very clear toxicity. Indeed, preclinical studies on FDG showed a LD_50_ of 600 mg/kg in mice and rats injected IP [55].

Furthermore, in clinical trials where 2DG was used to improve the efficacy of radiotherapy, 200–300 mg/kg of 2DG was administered orally after overnight fasting, resulting in significant side effects [56]. Therefore, 2DG and FDG at high concentration are not suitable for cancer diagnosis in humans and should be restricted to laboratory animals. However, in recent publication, proof-of-principle was provided that 2DG can be encapsulated in liposomes, which has the side effect of enhancing the CEST signal while potentially reducing the side effects of the agent for use in humans [57].

On the other side of the spectrum, glucosamine (GlcN) and *N*-Acetyl-d-glucosamine (GlcNAc) are two amino monosaccharides that are components of glycosaminoglycans, both of which are available over the counter as dietary supplements. Both can also be used as CEST contrast agents as they enter and accumulate in tumor cells via members of the GLUT family. The ability to image tumors by GlcN or GlcNAc CEST MRI has been demonstrated in several tumors of murine model [9, 14, 15]. The CEST detection of GlcN and GlcNAc allows the differentiation of tumors according to their aggressiveness. GlcN has also been shown to allow the detection of metastases, similar to FDG-PET [9]. The contribution of GlcN to CEST contrast can be attributed to GlcN itself and to its phosphorylated products. It can also be explained by tissue acidosis associated with lactate buildup [13, 15]. The NOE effect indicates that the CEST arises from intracellular GlcN and its metabolites, since free GlcN molecules do not elicit such effects [13, 15]. Another advantage of using GlcN or GlcNAc is that they can be detected for their amine/amide peaks around ~ 2–3 ppm, unlike other glucose analogs whose hydroxyl protons have a small chemical shift relative to the water signal (around 1–1.5 ppm). This raises the possibility that they can be detected with clinical scanners. GlcN is already available for clinical trials as it has an excellent safety profile, as evidenced by its widespread use as a dietary supplement.

#### Other non-metabolizable analogs

**Fig. 7:**
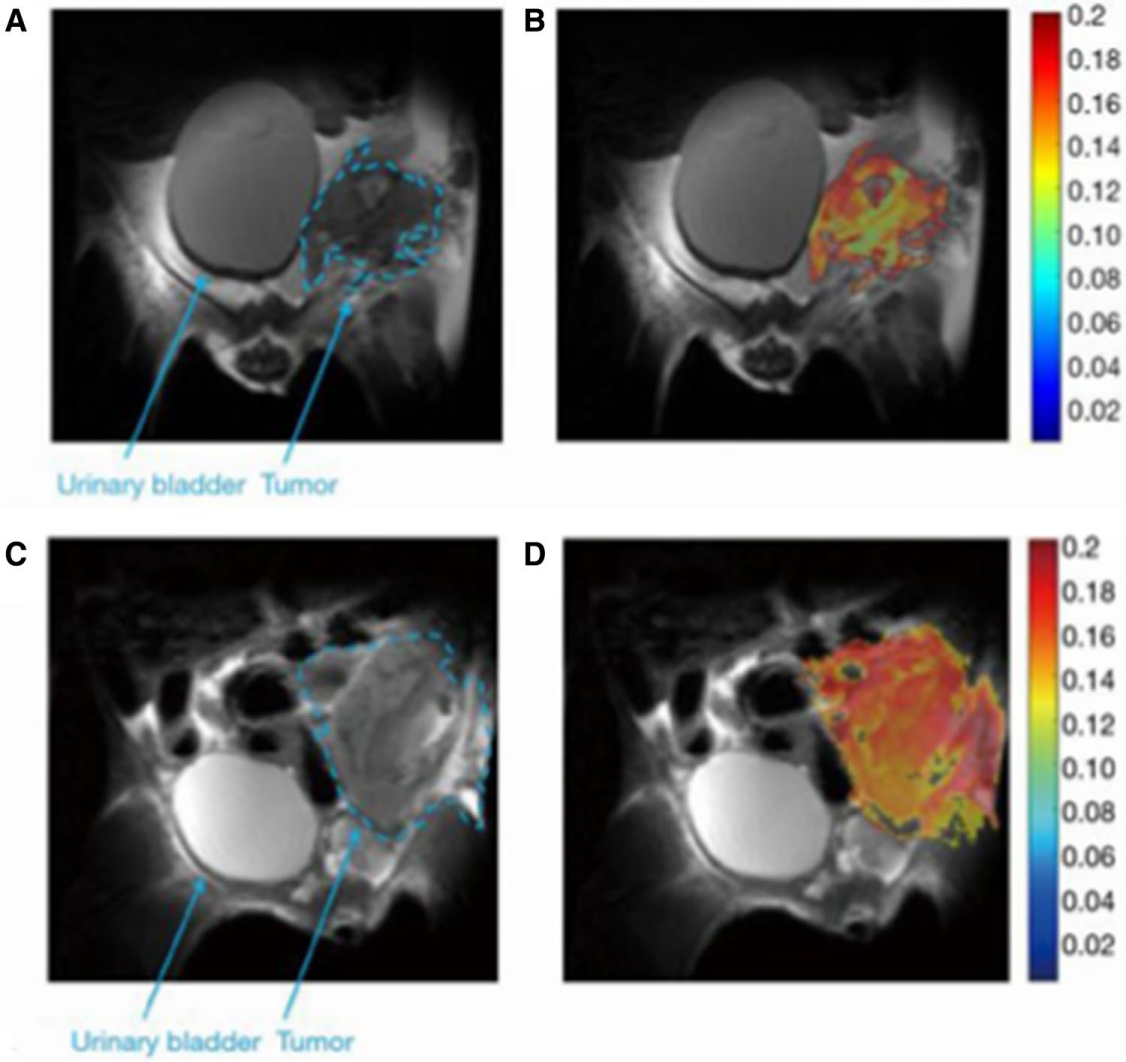
In vivo 2OMG and 6DG CEST MRI measurements in 4T1 tumors (7 T field). **A**, **C** T_2_-RARE anatomical images before administration of the agents. **B**, **D** MTR_asym_ images at 1.0 and 1.2 ppm following treatment with 2OMG (3 g/kg, IP) and 6DG (2.0 g/kg, IP), respectively, overlaid onto the T_2_ anatomical image. From [44], with permission

Other non-metabolized glucose analogs, such as 2-O-Methyl-D-glucose (2OMG) and 6-deoxy-D-glucose (6DG), were also tested to avoid risking the whole program on a single pair of molecules (Glc and 3OMG). These glucose analogs had to meet the following three criteria to be considered as potential CEST contrast agents: high uptake by tumors, high CEST signal or exchange-related effects, and very low or on toxic effects. Glucose analogs transported by GLUTs but not recognized by hexokinase have a low toxicity because they are not metabolized and are therefore excreted unchanged. Therefore, 2OMG and 6DG have shown promising results for imaging cancer cells using the CEST technique. Rivlin and Navon investigated and confirmed the inability of 2OMG and 6DG to undergo phosphorylation by 31P NMR spectroscopy studies of extracts from breast tumors (4T1 model) [44]. The Z-spectra of both 2OMG and 6DG were stable and unchanged over time [44], which can be explained by the fact that both agents are known to have a non-glycolytic metabolic profile. The sensitivity of 2OMG and 6DG in identifying regional differences is shown in Fig. 7. Therefore, it can be concluded that glucose analogs that are not phosphorylated by hexokinase are advantageous for cancer diagnosis using the CEST MRI technique.

In another set of experiments, GlcNAc was also compared to sucrose, owing to its high safety profile, as another alternative to Glc [14]. Both sucrose and GlcNAc provided a marked CEST effect that in contrast to Glc and 3OMG seemed to show a reduced dependency to pH. Interestingly, a remarkable CEST contrast was observed in two tumor murine models (breast and melanoma) up to 30 min post intravenous injection as demonstrated in Longo et al. [14]. The combination of good tumor CEST contrast enhancements and lack of toxicity makes sucrose, just as GlcNAc, as another potential interesting candidate contrast agent for tumor detection.

Finally, polymers of glucose units belonging to the class of plasma volume expanders, such as voluven (hydroxyethyl starch, 130 kDa) and dextran 70 (70 kDa), have also been explored as novel and clinically approved macromolecular CEST contrast agents by other groups [58–60]. They represent a novel platform based on the hydroxylic protons of the glucose moieties, although they were primarily exploited as blood pool MRI‐CEST agents to assess tumor vascularization upon increased accumulation and prolonged contrast enhancement in tumors.

## Translation of DGE MRI to clinics

### Development of novel imaging sequences and data postprocessing algorithms

For DGE MRI to be feasible in the clinics, a robust dynamic imaging is needed, as well as a pre-saturation with minimal direct water saturation. To achieve this, Zaiss et al. developed a 3D spiral centric reordered gradient echo (GRE) acquisition method called snapshot CEST MRI, and demonstrated the feasibility at 3 T and 9.4 T [61, 62]. Snapshot CEST imaging at 3 T consists of an adjustable saturation period and a subsequent readout phase of 2.5 s realized by a centric-spiral reordered 3D GRE readout. The snapshot sequence is a general readout and can be used for B_0_ and B_1_ mapping using a WASABI (simultaneous mapping of water shift and B1) preparation [21], CEST, spin-lock (SL), or T_1ρ_-weighted images as well as T_1_ mapping with a saturation recovery preparation. The phase information of the GRE also allows for online relative B_0_ inhomogeneity estimation and correction.

**Fig. 8:**
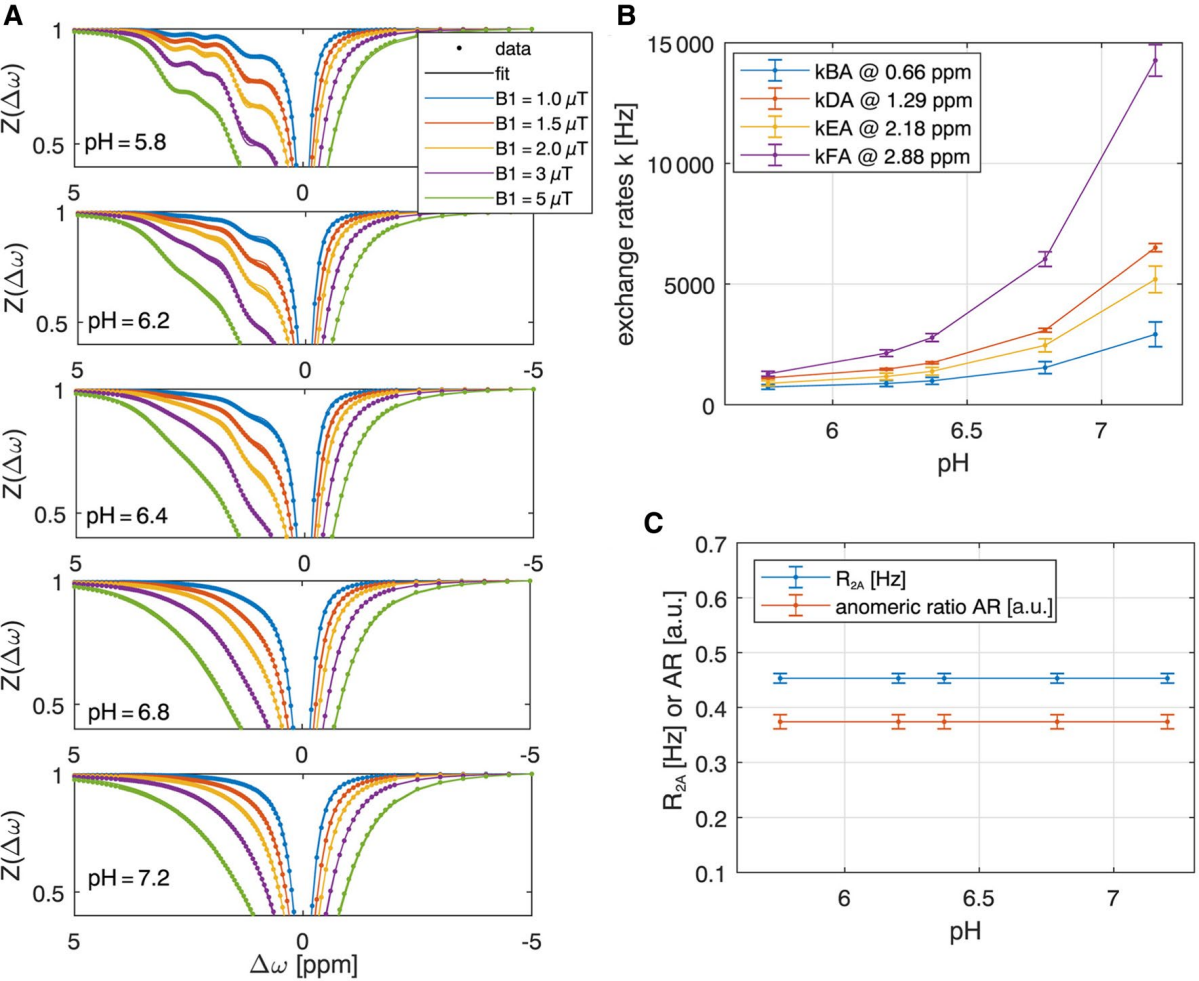
**A** Simultaneous multi‐B_1_‐pH‐fit of 25 Z‐spectra of 20 mM glucose model solutions acquired at 14.1 T yields glucose hydroxyl exchange rates as a function of pH at T = 37 °C **B** and R_2A_ and anomeric ratio **C**. From [48], with permission

**Fig. 9:**
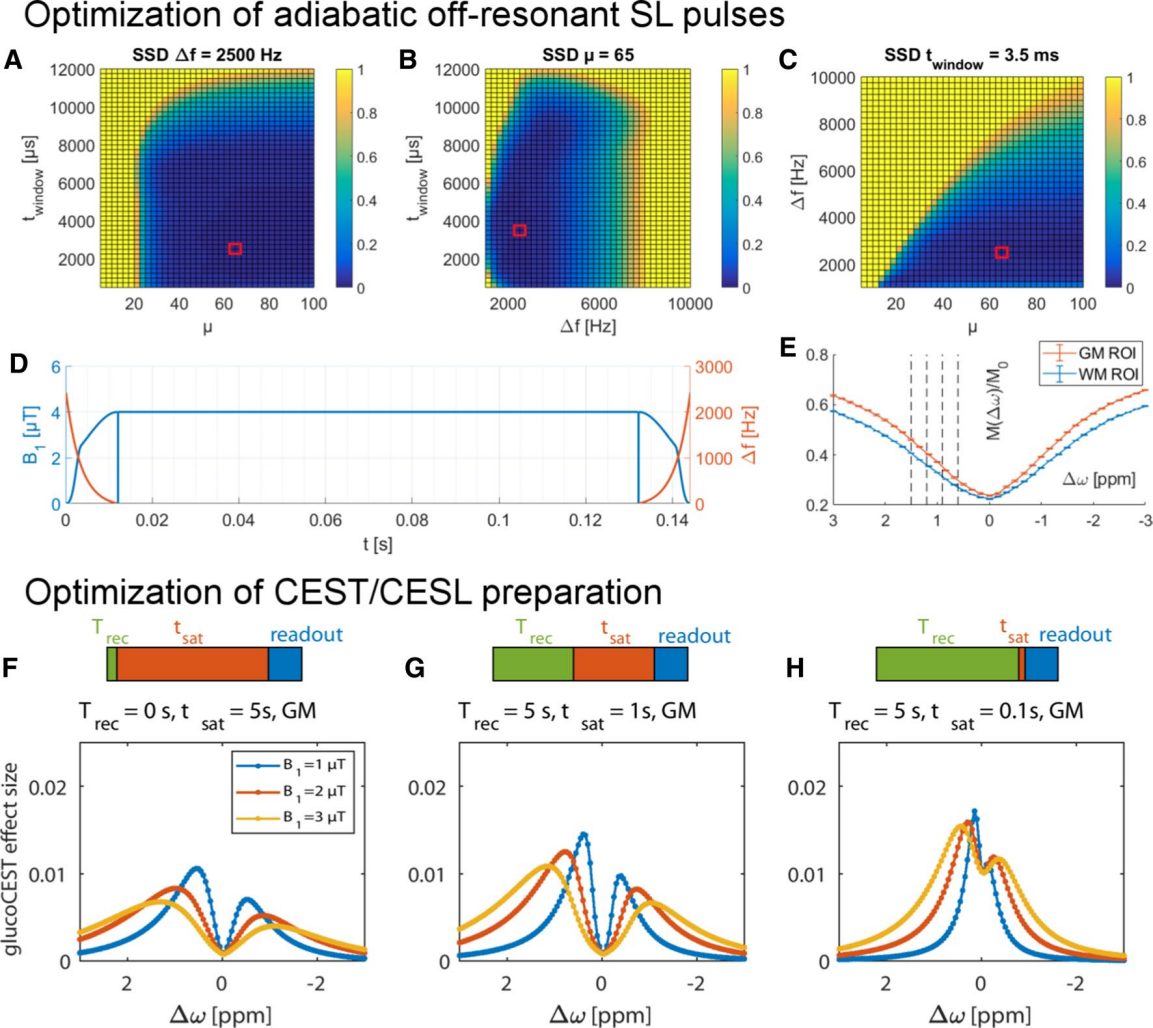
Optimization of the HSExp pulse for 3 T. **A**–**C** SSD to an analytical T_1ρ_ spectrum, which were used for pulse optimization [22]. Parameters resulting in a minimal SSD were ∆f = 2.5 kHz, µ = 65, and t_window_ = 3.5 ms, marked with a red square. **D** The SL cluster at 0 ppm for a TSL = 120 ms. **E** Measured Z‐spectra and their standard error for this pulse cluster in a WM and GM ROI. DGE_ρ_ offsets acquired in the dynamic experiment are marked with a dashed line (0.6, 0.9, 1.2, and 1.5 ppm). Simulated DGE_ρ_ effect after d-glucose injection (**F**–**H**) in the steady‐state CEST regime (**F**), the intermediate regime with only one second of saturation (**G**), and the SL regime with 100 ms of saturation (**H**). From [22] and [48], with permission

To make the best choice for both pre-saturation and MR readout of a DGE MR sequence, a simulation for the estimation of CEST signal intensities for different setups is needed. It was realized using an N-pool Bloch-McConnell equations with and without RF irradiation. A CEST sequence simulation was thus conducted and a fitting framework, based on a newly developed analytical CEST theory [48, 63–65], was applied for verification [48]. The simulation framework, postprocessing scripts, and sample data can be found in an online repository (https://www.cest-sources.org). The individual exchange rates of the glucose individual hydroxyl groups under in vivo conditions were estimated by a Bloch-McConnell least-squares fit to multi-pH and multi B_1_ data acquired in glucose solutions at 14.1 T. At pH = 7.2 and *T* = 37 °C the exchange rates of 1200 Hz (0.66 ppm), 2997 Hz (1.3 ppm), 3543 Hz (2.1 ppm), and 5863 Hz (2.88 ppm) were obtained for the different Glc hydroxyl groups resonating at different frequencies (Fig. 8). Based on these results, the optimal recovery delay and pre-saturation parameters in terms of contrast-to-noise ratio could be determined by Bloch-McConnell simulations including tissue-like direct water saturation and semi-solid magnetization transfer. For CESL or DGE_ρ_ at 3 T the best pre-saturation in tissue was achieved by short but strong saturation (see Fig. 9F–H), realized by one SL pulse of duration 100 ms and B_1_ = 4 μT at 0.4 ppm. The results for higher field strength (9.4 T) were similar, with slightly higher power for CEST pre-saturation (5 µT) and a maximum signal at 0.25 ppm.

For optimization and robustness against B_1_ and B_0_ inhomogeneities, HSExp adiabatic SL pulses [66] were optimized for the use at 3 T. Based on the requirements for optimal saturation parameters obtained by the Bloch-McConnell fitting [64], a T_1ρ_-weighted SL imaging with an optimal locking power of 4 µT was set up at 3 T with a locking time TSL = 120 ms after 4 s of relaxation.

**Fig. 10:**
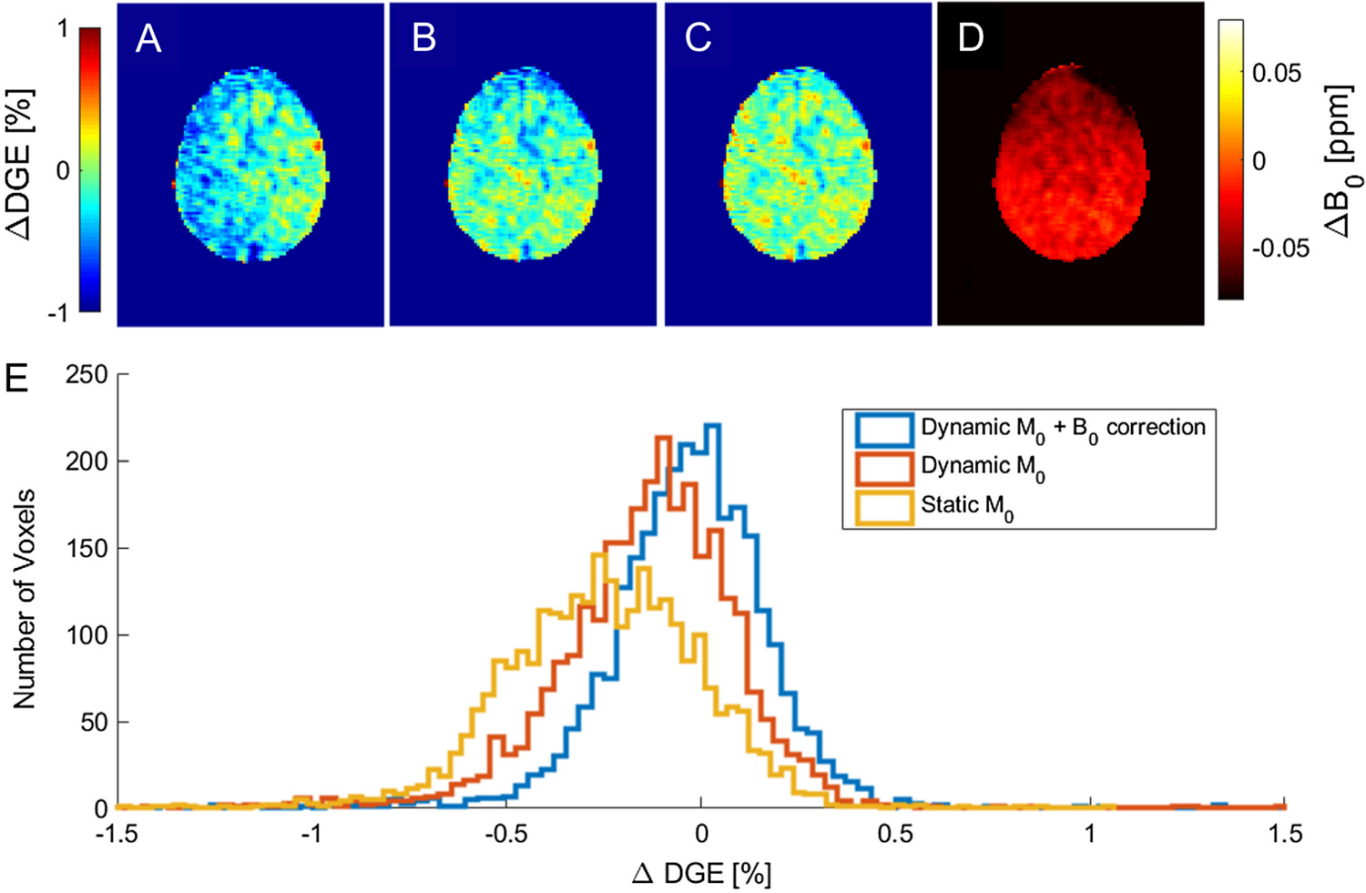
Effect of motion correction, interleaved M_0_, and dynamic B_0_ correction. **A** ∆DGE_ρ_ [%] map of a volunteer measurement (image number 16 at ∆ω = 0.9 ppm) after motion correction. Here, the entire left hemisphere seems to be affected by a strong hypointensity. **B** ∆DGE_ρ_ map with a normalization to the corresponding image at − 300 ppm. Here, the image is more flat, but still shows slight correlation with B_0_ (**D**), especially in the anterior part. **C** ∆DGE_ρ_ map with the same normalization as (**B**) and an additional B_0_ correction as proposed in Windschuh et al. [68]. The dynamic B_0_ correction normalizes the anterior part that is hypointense in (**B**). **D** ∆B_0_ map [ppm] relative to the beginning of the measurement. **E** Histogram for the images in (**A**–**C**). From [22], with permission

As motion and B_0_ shifts during the dynamic measurement can lead to artifacts [67], the post-processing of DGE_ρ_ MRI data consisted of the following steps: (I) optimized retrospective rigid body motion correction, (II) dynamic normalization by repetitive M0 scans at − 300 ppm, and (III) dynamic B_0_ correction using the phase information from the GRE readout [68]. The impact of steps (II) and (III) on the data is shown in Fig. 10.

### mDGE imaging in body

**Fig. 11:**
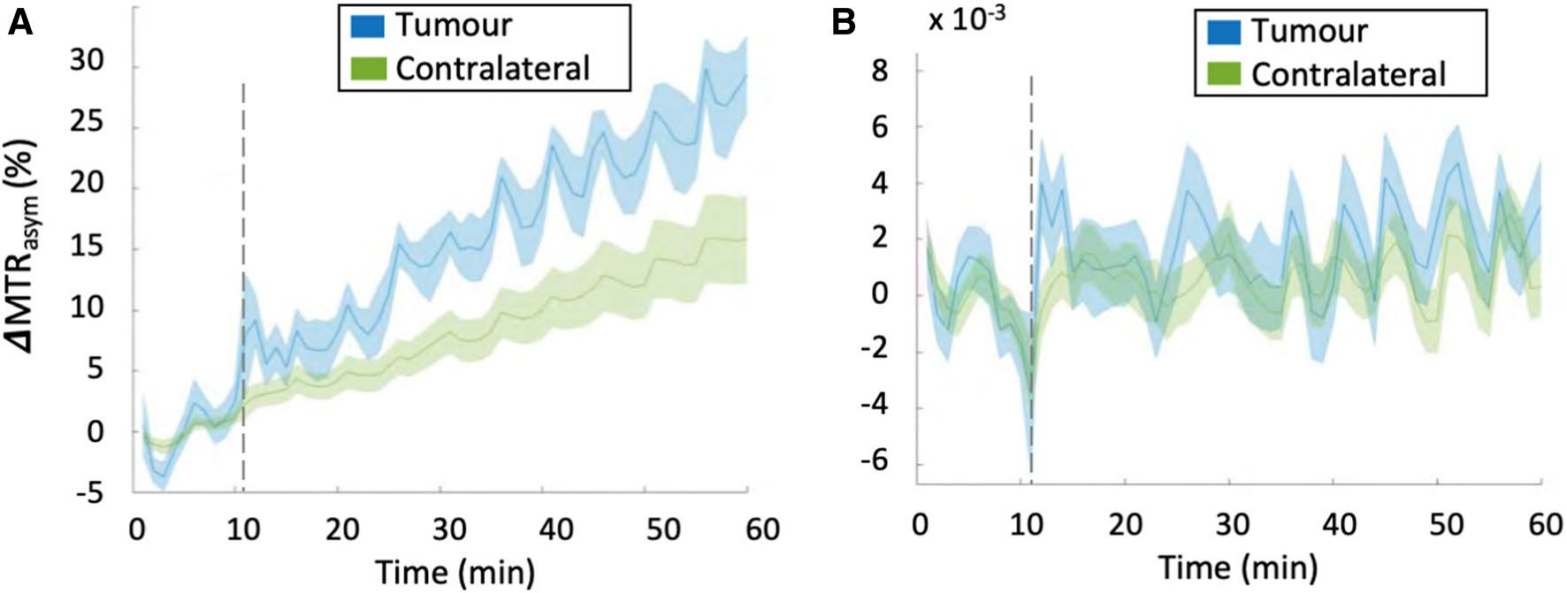
**A** MTR_asym_ signal integrated in the range of 2—3 ppm before B_0_ and B_1_ correction shows field drifts both in tumor and contralateral regions of a patient with prostate cancer. It is worthwhile to note that the changes due to B_0_ drift are much larger in the body, due to the increased drift observed. In this case the B_0_ drifts across slice and entire scan duration were found to be 25 Hz (0.2 ppm) and 200 Hz (1.56 ppm), respectively. **B** After B_0_ correction, no significant enhancement in MTR_asym_ signal is observed and the signal intensity is significantly reduced. From [25], with permission

To date, only a few studies have been reported for mDGE MRI outside the brain at 3 T [24–26], while several groups have demonstrated signal changes of mDGE MRI in human brain, following the injection of Glc at 3 T and 7 T [17–23]. The signal responses to Glc injection in human body among a few studies were irregular as the methods used for each mDGE MRI study were diverse in terms of CEST acquisition, as well as B_0_ shift and B_1_^+^ correction. Additionally they may be due to numerous aspects of the experiment, including a combination of differences in injection protocol in addition to variations in the metabolic response of individual patients, as well as the inherent low SNR and sensitivity to motion [67]. To better understand the signal variability from the different mDGE MRI studies in human body applications, Kim et al. [25] optimized mDGE MRI protocols based on rapid assessment of B_0_ and B_1_^+^ and sought to characterize the mDGE MRI signal in two types of cancer with varying expected metabolic rates and blood volume. Additionally, an infusion protocol for intravenous (i.v.) 20% Glc was optimized using a hyperglycemic clamp to maximize the likelihoods of detecting the mDGE MRI signal, by separating measurement of perfusion from metabolism. Results showed that B_0_ inhomogeneity leading to a shift in the Z-spectra affects the magnitude of mDGE signal over time. In addition, the results suggest that motion correction in addition to B_0_ is crucial to avoid mistaking mDGE MRI signal changes for a Glc response while B_0_ field drift remained a significant contributor. Finally, in spite of all these optimizations, no significant mDGE MRI signal was observed in tumor regions of any patients with lymphoma and prostate cancer at 3 T after B_0_ field drift correction (Fig. 11). Therefore, it has been concluded that mDGE MRI signal at 3 T remains elusive in human body regions, where detection of the originally small mDGE MRI signal is difficult due to physiological movements and substantial effects of B_1_^+^ and B_0_.

### DGE and DGE_ρ_ imaging in brain

Contrary to body applications, early experience shows that DGE and DGE_ρ_ signal detection in human brain seem possible at 3 T [22, 23] if appropriate post-processing steps, i.e., corrections for motion-related artifacts are applied. Although the effect size was small, generated DGE_ρ_ maps showed unique patterns, partially correlated with the gadolinium (Gd)-based T_1_ tumor ring enhancement. In a study of 3 glioma patients, a significant glucose uptake could be observed with a DGE_ρ_ effect strength in the range of 0.5% of the water signal for the patients with BBB breakdown. Statistical evidence can decrease when uncorrected motion is included as a thorough analysis revealed.

**Fig. 12:**
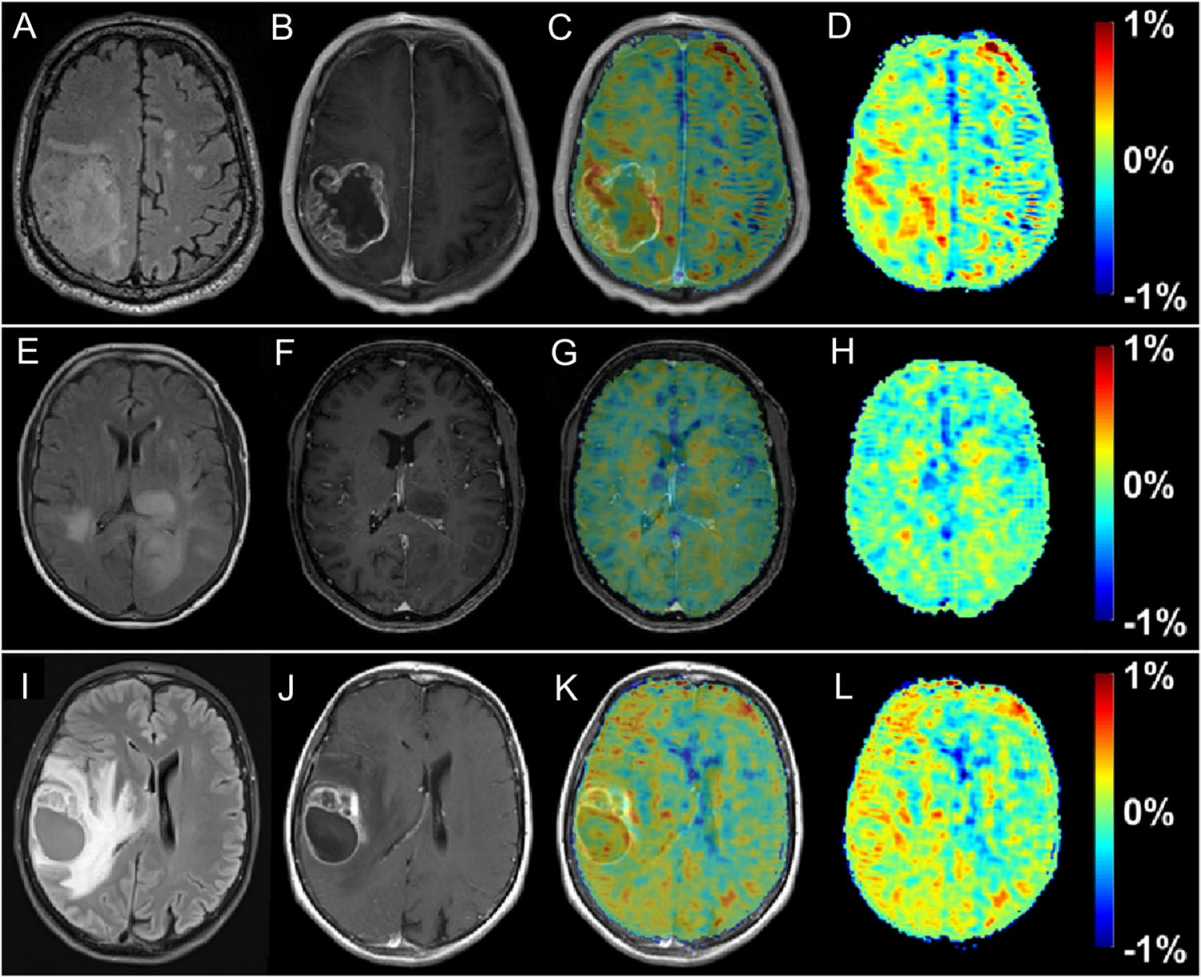
T_2_ FLAIR (**A, E, I**), T_1_‐ce (**B**,**F**,**J**), T_1_‐ce with overlaid ∆DGE_ρ_ map (**C, G, K**), and the ∆DGE_ρ_‐maps (**D**,**H**,**L**) of patient 1 (**A**–**D**, ~ 7 min post-injection), 2 (**E**–**H**, ~ 7 min post-injection), and 3 (**I**–**L**, ~ 9 min post-injection). From [22], with permission

Compared to reported effects at 7 T in humans of 2–6% [17, 19], the observed DGE_⍴_ effect size of ~ 0.5% at a clinical field strength of 3 T is small, but consistent with Bloch simulations, where the effect was calculated to be two to three times smaller than at 7 T. The maximum effect size was observed approximately 8 to 9 min after beginning of glucose injection in general, which is in good agreement with the literature on animal experiments [69]. The different contributions to the signal, especially vascular and intracellular contributions are still discussed in the literature. On the contrary, there is a consent in the community that glucose leaking through a damaged BBB into the extravascular, extracellular space contributes to a large extent to the glucoCEST signal. Therefore, a correlation between Gd contrast-enhanced images and DGE_⍴_ images is expected as Gd-based agents would also enter this space via a BBB breakdown. The following observations were made further [70]: first, when a visible strong or faint BBB breakdown can be observed, in general a positive DGE_⍴_ signal was also detected. However, spatially, there was only a rough match with the tumor region indicated by the Gd enhancement, i.e., between the hyper-intensity patterns in the DGE_⍴_ and the DCE contrast. The observed hyper-intense areas in the DGE_⍴_ maps might originate primarily from higher glucose levels or lower pH values attributed to higher tumor activity. However, it is impossible to define at this stage what the main source of signal enhancement is. Such a conclusion requires a larger dataset if possible with a higher signal to noise ratio and higher contrast to noise ratio for DGE_⍴_. At the moment, the relation between DCE and DGE_⍴_ cannot be fully described and one cannot rule out that some of the differences are due to the lower sensitivity of DGE_⍴_ compared to DCE (Fig. 12).

Finally, in general, the current signal to noise ratio (SNR) renders the possibility to analyze the signal changes over time through the use of a pharmacokinetic (pK) model, as originally proposed in the GLINT application, very unlikely, unless a solution to boost the SNR can be found, which could, inter alia, be the exclusive use of this contrast at higher field strength.

## Future direction

### Implementation into clinical practice

One of the main conclusions that can be drawn from most studies so far is that the signal of DGE and DGE_⍴_ MRI is generally small, and thus very sensitive to motion artifacts. If motion is not too severe it can be corrected, and a reliable contrast can be generated. If at all, then deeper insight can be gained at 7 T, and we would also generally recommend aiming for 7 T to be used for further applications of DGE and DGE_⍴_ MRI due to the increase in both CNR (contrast to noise ratio) and SNR. Pre-saturation using pulsed adiabatic spinlock was very close to the ideal CW (continuous wave)-CEST case. However, other groups reported that this can be outperformed using on-resonant variable delay multi-pulses, which should be considered and compared to the approach reported here [71].

Currently, the response from brain tumor patients seems variable and the small number of patients scanned does not allow to provide a final clinical picture. However, there seem to be a subtle increase in the DGE and DGE_⍴_ signal over time which, if not overshadowed by motion artifacts, should provide further insights into the biological characterization of brain tumors.

As motion can severely impact the reliability, whole-brain snapshot approaches with prospective motion correction and B_0_, B_1_, and motion navigator scans for each CEST acquisition would be ideal. This being said, DGE and DGE_⍴_ MRI outside the brain might be achievable in the near future, for instance, breast applications, where motion and B_0_ are not too severe, but in other parts of the body, motion artifacts have to be under control first to achieve a required temporal SNR in the order of 100:1 to be able to detect any signal at all.

Finally, one of the biggest issues to be addressed is possibly the actual risks based on the injection of a large bolus of a high volumetric concentration of Glc, which might, among others, lead to venous thrombosis or thrombophlebitis [72].

### Potential diagnostic benefits

As observed from the presented data, the DGE and DGE_⍴_ signal detected so far in patients at 3 T resembles DCE increases in areas of broken-down BBB. Based on the preclinical data shown in this review, it is, however, still unclear whether DGE and DGE_⍴_ signal might also shed a light on tumor basal metabolism, expression of tumor markers, or the metabolic alteration induced by constitutive activation of oncogenes in tumor development. If the results from animal models can be transferred into clinical practice, DGE and DGE_⍴_ MRI could be a promising tool for evaluation of treatment response in patients with gliomas and brain metastases, especially in light of new therapies based on immunomodulation and oncolytic viruses. However, the current state of the art shows that work is needed to improve the specificity and detection power at clinical field strengths.

## Conclusion

Through a thorough review of the literature on applications of glucoCEST/CESL so far, one can draw several conclusions. First, it is now clear that Glc can be detected in vivo, and may provide a very unique signature in primary brain cancer (glioma) patients. Several glucose analogs can also be detected and have shown promising results in preclinical settings. Then, several non-metabolizable glucose derivatives (among which 3OMG) have been tested and showed promising early results for translation into the clinics. 3OMG in particular also showed comparable imaging results as the gold standard FDG-PET in animals, thereby highlighting its intracellular contribution (being taken up, yet not phosphorylated), and might be considered therefore as a prime candidate for first-in-man studies. Finally, at this point, further translation into body oncological applications remain limited by the small signal and large number of confounding factors present, e.g., B_0_ and B_1_ inhomogeneities, as well as elevated motion artifacts.
